# Effects of Amoxicillin and Augmentin on Cystine-Glutamate Exchanger and Glutamate Transporter 1 Isoforms as well as Ethanol Intake in Alcohol-Preferring Rats

**DOI:** 10.3389/fnins.2016.00171

**Published:** 2016-04-25

**Authors:** Alqassem Y. Hakami, Alaa M. Hammad, Youssef Sari

**Affiliations:** Department of Pharmacology and Experimental Therapeutics, College of Pharmacy and Pharmaceutical Sciences, University of ToledoToledo, OH, USA

**Keywords:** GLT-1 isoforms, xCT, GLAST, amoxicillin, augmentin

## Abstract

Alcohol dependence is associated with alteration of glutamate transport and glutamate neurotransmission. Glutamate transporter 1 (GLT-1) is a major transporter that regulates the majority of extracellular glutamate concentration, which is also regulated by cystine-glutamate exchanger (xCT). Importantly, we recently reported that amoxicillin and Augmentin (amoxicillin/clavulanate) upreglulated GLT-1 expression in nucleus accumbens (NAc) and prefrontal cortex (PFC) as well as reduced ethanol consumption in male P rats. In this study, we examined the effects of amoxicillin and Augmentin on GLT-1 isoforms (GLT-1a and GLT-1b), xCT, and glutamate/aspartate transporter (GLAST) expression in NAc and PFC as well as ethanol intake in male P rats. We found that both compounds significantly reduced ethanol intake, and increased GLT-1a, GLT-1b, and xCT expression in NAc. However, only Augmentin increased GLT-1a, GLT-1b, and xCT expression in PFC. There were no effects of these compounds on GLAST expression in NAc and PFC. These findings demonstrated that Augmentin and amoxicillin have the potential to upregulate GLT-1 isoforms and xCT expression, and consequently attenuate ethanol dependence.

## Introduction

Glutamate neurotransmission has a critical role in alcohol dependence and other drugs of abuse, which involving several mesocorticolimbic brain regions, including nucleus accumbens (NAc) and prefrontal cortex (PFC) (Goldstein and Volkow, 2002; Obara et al., 2009; Das et al., 2015). PFC is a brain region that was suggested to regulate cognitive and emotional processes and triggers drug seeking (Goldstein and Volkow, 2002), and the NAc has long been implicated in goal-directed drug seeking behavior (Childress et al., 1999). Glutamate appears to be the primary driver of PFC neurons, and drug seeking requires the release of this glutamate from the PFC to the NAc (Childress et al., 1999; Goldstein and Volkow, 2002; Shalev et al., 2002; Capriles et al., 2003; McFarland et al., 2003). Therefore, we tested the effects of amoxicillin and Augmentin on certain glutamate transporters in NAc and PFC. Glutamate transporter 1 (GLT-1, its human homolog is excitatory amino acid transporter 2, EAAT2) is a key transporter that regulates the majority of extracellular glutamate concentration in the brain (Rothstein et al., 1996; Tanaka et al., 1997; Danbolt, 2001). Importantly, studies from our laboratory revealed that chronic ethanol intake reduced GLT-1 expression and increased the extracellular glutamate concentration in NAc (Das et al., 2015). This study also demonstrated that treatment with ceftriaxone, β-lactam antibiotic known to upregulate GLT-1, reversed the effects of ethanol exposure. Furthermore, we reported that male P rats administered Augmentin or amoxicillin showed reduction in ethanol consumption, which was associated in part with upregulation of GLT-1 expression in PFC and NAc (Goodwani et al., 2015).

In the present study, we focused our investigation on GLT-1 isoforms (GLT-1a and GLT-1b) and other glial glutamate transporters such as cystine-glutamate exchanger (xCT) and glutamate/aspartate transporter (GLAST, its human homolog is EAAT1). The focus on investigating GLT-1 isoforms was based on the fact that these proteins are differentially expressed according to a disease model. For instance, patients with amyotrophic lateral sclerosis were found to have upregulated GLT-1b expression and downregulated GLT-1a expression in motor cortex (Maragakis et al., 2004). In addition, GLT-1a is expressed in neurons and astrocytes, however, GLT-1b is expressed mainly in astrocytes (Berger et al., 2005; Holmseth et al., 2009). The difference in their ability to regulate extracellular glutamate concentration has not been confirmed yet. Thus, the present study investigated for the first time the effects of chronic ethanol exposure on the expression of GLT-1 isoforms. We also investigated xCT, which is another glial glutamate transporter that was also shown to regulate extracellular glutamate concentration (Baker et al., 2002; Moran et al., 2005). xCT exchanges internal glutamate for external cystine with the electrochemical gradient (Volterra et al., 1996; Danbolt, 2001). In addition, xCT is co-expressed with GLT-1 in astrocytes to regulate extracellular glutamate concentration. Decrease of xCT expression can affect extracellular glutamate concentration, which can lead to loss of glutamatergic tone on presynaptic mGluR2/3, thereby causing an increase in synaptic glutamate release (Moran et al., 2005). Finally, we investigated the effects of ethanol exposure as well as Augmentin and amoxicillin treatments on GLAST expression in PFC and NAc.

## Materials and methods

### Animals

Male P rats were obtained from Indiana University, School of Medicine (Indianapolis, IN, USA) at the age of 21–30 days, and housed in the Department of Laboratory Animal Resources, University of Toledo, Health Science Campus. At the age of 90 days, rats were individually housed in a plastic corn-cob bedding tubs and had a free access to food and water ad lib. The room temperature was maintained at 21°C and 50% humidity with a 12-h light-dark cycle to resemble the natural habitat throughout the experiment procedures. All animal procedures were in compliance and approved by the Institutional Animal Care and Use committee of The University of Toledo in accordance with the guidelines of the Institutional Animal Care and Use Committee of the National Institutes of Health and the Guide for the Care and Use of Laboratory Animals.

### Behavioral drinking paradigms

Male P rats were exposed to free choice of two ethanol concentrations, 15 and 30%, (except for the ethanol naïve (water) group), water and food for a period of 5 weeks. After the third week of drinking paradigm, ethanol and water intakes were measured three times a week for 2 weeks and used as a baseline. The ethanol and water intake measurements were expressed as g/kg of body weight/day. Animals with a baseline ethanol intake of less than 4 g/kg/day were not included in the study as it was adopted in previous studies (Sari et al., 2011, 2013; Sari and Sreemantula, 2012). At the age of 3 months, rats were randomly divided into four different groups: (a) Ethanol naïve group, which had a free access to water and food only for 5 weeks, and received five consecutive daily i.p. injections of saline vehicle solution on Week 6 (*n* = 6); (b) Ethanol-vehicle (saline) group, which received five consecutive daily i.p. injections of saline vehicle solution on Week 6 (*n* = 6); (c) Ethanol-amoxicillin group, which received five consecutive daily 100 mg/kg, i.p. injections of amoxicillin on Week 6 (*n* = 6); and (d) Ethanol-Augmentin group received five consecutive daily 100 mg/kg, i.p. injections of Augmentin on Week 6 (*n* = 6).

### Brain tissue harvesting

Rats were euthanized 24 h after the last i.p. injections of saline or drugs using carbon dioxide and directly decapitated with guillotine. Brains were then immediately placed on dry ice and stored at −80°C. Brain regions (PFC and NAc) were dissected according to the Rat Brain Atlas (Paxinos and Watson, 1997) using cryostat apparatus set at −20°C. Extracted brain regions were then stored at −80°C for western blot analysis.

### Western blot protocol for detection of GLT-1 isoforms, xCT, and GLAST

Brain regions were lysed using regular lysis buffer as described in recent work (Sari et al., 2011). Protein transfer was performed on a PVDF membrane using gel electrophoresis method (Bio-Rad, Hercules, CA). Then, membranes were blocked with 3% milk in TBST (50 mM Tris HCl; 150 mM NaCl, pH 7.4; 0.1% Tween 20) for 30 min at room temperature. Moreover, membranes were incubated overnight at 4°C with one of the following primary antibodies: rabbit anti-GLT-1a (1:5000; gift from Dr. Jeffery Rothstein), rabbit anti-GLT-1b (1:5000; gift from Dr. Paul Rosenberg), rabbit anti-xCT antibody (1:1000; Abcam), and rabbit anti-EAAT1 (GLAST) antibody (1:5000; Abcam). Mouse anti β-tubulin was used as loading control (1:5000; Cell signaling technology). On the next day, membranes were washed with TBST for five times and then blocked with 3% milk in TBST for 30 min. Membranes were then incubated with secondary antibodies (anti-rabbit 1:5000, Thermo scientific and anti-mouse 1:5000, Cell signaling technology) for 90 min at room temperature. Membranes were incubated with the SuperSignal West Pico Chemiluminescent substrate and further exposed to Kodak BioMax MR Film (Fisher Inc.); and films were developed on SRX-101A machine. MCID system was used for Western blot analysis, and the results were presented as a percentage of the ratio of tested protein/β-tubulin, a control loading protein. The data from ethanol naïve groups were reported as 100% to evaluate the changes in protein expression in brain regions following chronic ethanol consumption. In addition, the data from ethanol vehicle group were reported as 100% to evaluate the changes in protein expression in brain regions as compared to β-lactam-treated groups.

### Statistical analyses

Two-way Mixed ANOVA followed by Bonferroni multiple comparisons was conducted to determine the main effect of Day or Treatment or Day x Treatment interaction on the average daily ethanol and water intakes. One-way ANOVA followed by Dunnett's *post-hoc* test was used to determine the effects of amoxicillin and Augmentin treatments on ethanol and water intakes in each day. An independent *t*-test was used to analyze Western blot data between ethanol naïve and ethanol-vehicle groups. In addition, one-way ANOVA followed by Newman-Keuls *post-hoc* test was used to compare Western blot data from treatment groups (amoxicillin and Augmentin) and control group (ethanol-vehicle). Western blot densities from control groups (ethanol naïve or ethanol vehicle) were converted to 100%. All statistical analyses were based on a *p* < 0.05 level of significance.

## Results

### Effects of amoxicillin and Augmentin treatments on ethanol and water intake

**Figure 1:**
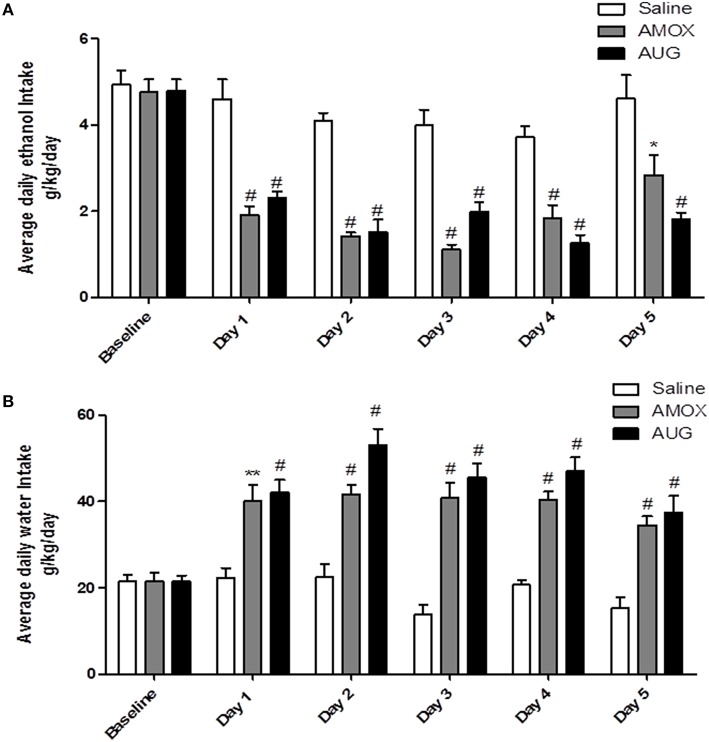
**(A)** Effects of amoxicillin (AMOX) and Augmentin (AUG) treatments on average daily ethanol intake (g/kg/day) as compared to ethanol vehicle. **(B)** Effects of amoxicillin and Augmentin treatments on water consumption (g/kg/day) as compared to ethanol vehicle. Data are represented as mean ± SEM; (^*^p < 0.05; ^**^p < 0.01; ^#^*p* < 0.001), (*n* = 6 for each group).

Two-way (mixed) ANOVA followed by Bonferroni multiple comparisons demonstrated a significant main effect of Day [*F*_(1, 5)_ = 31.92, *p* < 0.0001] and a significant Day x Treatment interaction [*F*_(2, 10)_ = 4.559, *p* < 0.0001] of ethanol intake. Moreover, one-way ANOVA followed by Dunnett's *post-hoc* test revealed a significant decrease in ethanol intake in amoxicillin- and Augmentin-treated groups as compared to saline-treated group from Day 1 through Day 5 (*p* < 0.001), except on Day 5 for amoxicillin group *p* < 0.05 (Figure 1A). In addition, statistical analysis revealed a significant main effect of Day [*F*_(1, 5)_ = 15.88, *p* < 0.0001] and a significant Day x Treatment interaction [*F*_(2, 10)_ = 5.285, *p* < 0.0001] of water consumption. One-way ANOVA followed by Dunnett's *post-hoc* test demonstrated a significant increase in water consumption in amoxicillin- and Augmentin-treated groups as compared to saline-treated group from Day 1 through Day 5 (*p* < 0.001) except on Day 1 for amoxicillin group *p* < 0.01 (Figure 1B).

### Effects of chronic ethanol consumption on GLT-1 isoforms (GLT-1a and GLT-1b), xCT, and GLAST expression in NAc and PFC

**Figure 2:**
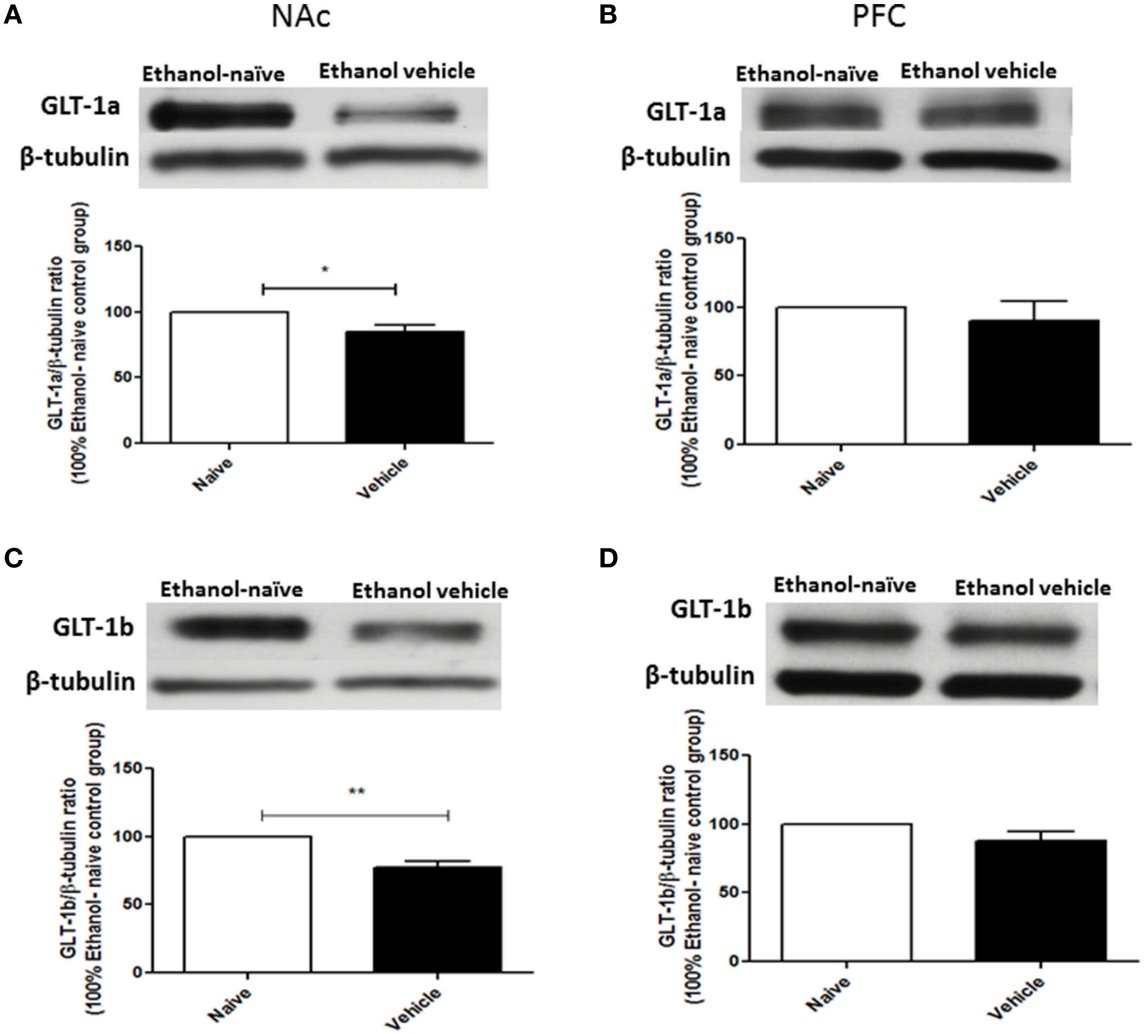
**Effect of chronic ethanol consumption on GLT-1 isoforms (GLT-1a and GLT-1b) expression in NAc and PFC. (A)** Statistical analysis showed significant downregulation of GLT-1a expression in ethanol vehicle group as compared to ethanol naïve (water) group in NAc. **(B)** Statistical analysis showed no significant difference of GLT-1a expression in PFC between ethanol naïve and ethanol vehicle groups. **(C)** Statistical analysis revealed significant downregulation of GLT-1b expression in ethanol vehicle group as compared to ethanol naïve group in NAc. **(D)** Statistical analysis also revealed no significant difference of GLT-1b expression in PFC between ethanol naïve and ethanol vehicle groups. Data are shown as mean ± SEM; (^*^p < 0.05, ^**^p < 0.01); (*n* = 5 for each group).

Using Western blot, we investigated the effects of chronic ethanol consumption on GLT-1a and GLT-1b expression in NAc and PFC. Statistical analysis using independent *t*-test demonstrated a significant downregulation of GLT-1a (Figure 2A, *p* < 0.05) and GLT-1b (Figure 2C, *p* < 0.01) expression in ethanol vehicle group as compared to ethanol naïve group in NAc, However, statistical analysis using independent *t*-test did not reveal any changes in GLT-1a (Figure 2B) and GLT-1b (Figure 2D) expression in PFC.

**Figure 3:**
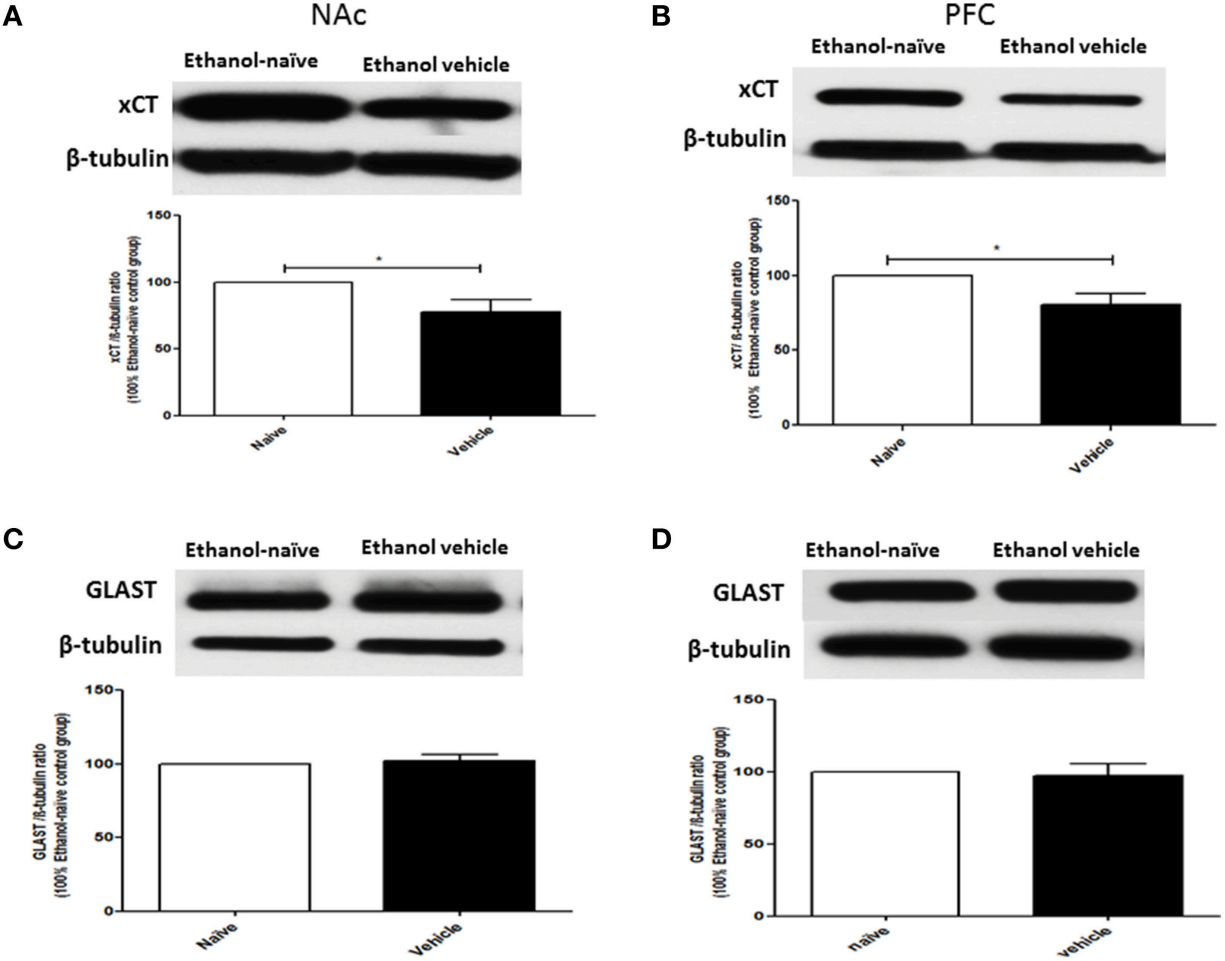
**Effects of chronic ethanol consumption on xCT and GLAST expression in NAc and PFC. (A)** Statistical analysis showed significant downregulation of xCT expression in ethanol vehicle group as compared to ethanol naïve group in NAc. **(B)** Statistical analysis showed significant downregulation of xCT expression in ethanol vehicle group as compared to ethanol naïve group in PFC. **(C)** Statistical analysis revealed no significant difference of GLAST expression in NAc between ethanol naïve and ethanol vehicle groups. **(D)** Statistical analysis also revealed no significant difference of GLAST expression in PFC between ethanol naïve and ethanol vehicle groups. Data are shown as mean ± SEM; (^*^p < 0.05); (*n* = 5−6 for each group).

The effects of chronic ethanol consumption on xCT and GLAST expression in NAc and PFC were further investigated between ethanol vehicle and ethanol naïve groups. Statistical analysis using independent *t*-test showed a significant downregulation of xCT expression in ethanol vehicle group as compared to ethanol naïve group in NAc (Figure 3A; *p* < 0.05) and PFC (Figure 3B; *p* < 0.05). However, statistical analysis did not reveal any significant difference in GLAST expression between ethanol vehicle and ethanol naïve groups in NAc (Figure 3C) and PFC (Figure 3D).

### Effects of amoxicillin and Augmentin on GLT-1 isoforms (GLT-1a and GLT-1b), xCT, and GLAST expression in NAc and PFC

**Figure 4:**
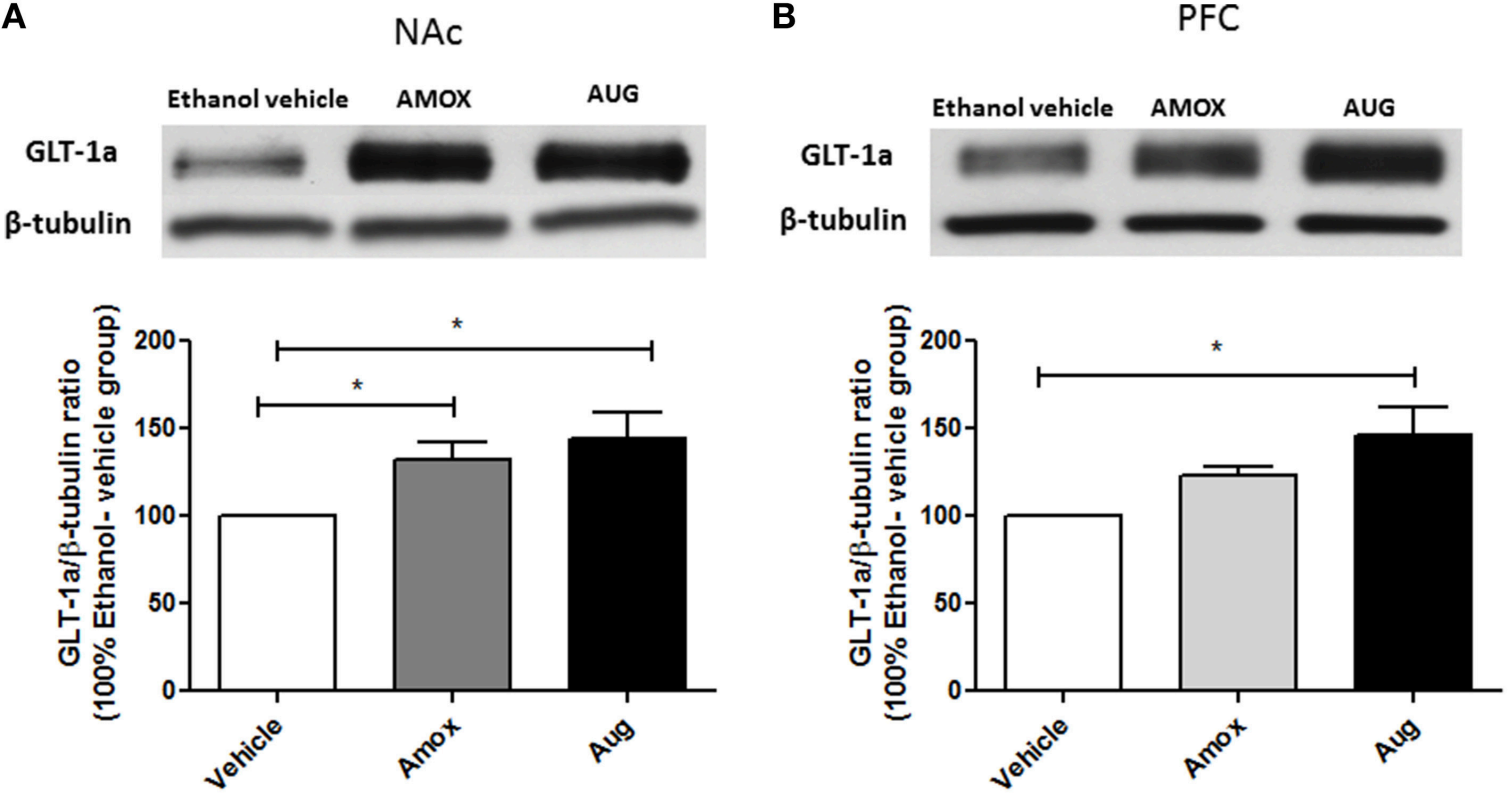
**Effects of Amoxicillin (AMOX) and Augmentin (AUG) on GLT-1a expression in NAc and PFC. (A)** Statistical analysis showed significant upregulation of GLT-1a expression in NAc in both amoxicillin and Augmentin groups compared to ethanol vehicle (saline) group. **(B)** Statistical analysis revealed significant upregulation of GLT-1a expression in PFC in Augmentin-treated group compared to ethanol vehicle group. However, no significant difference of GLT-1a expression was observed between amoxicillin treated group and ethanol vehicle group. Data are shown as mean ± SEM, (^*^p < 0.05); (*n* = 5 for each group).

**Figure 5:**
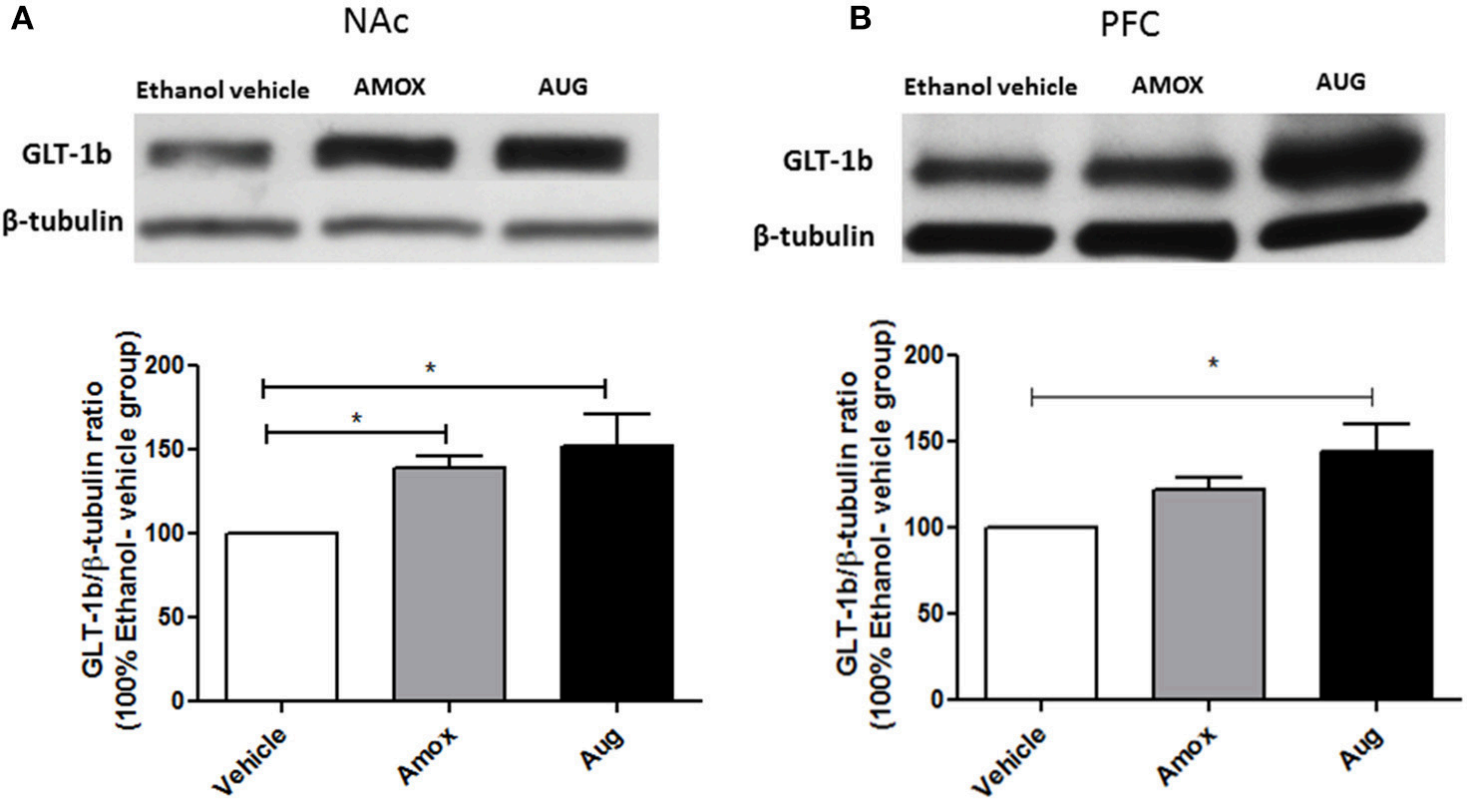
**Effects of amoxicillin (AMOX) and Augmentin (AUG) on GLT-1b expression in NAc and PFC. (A)** Statistical analysis showed significant upregulation of GLT-1b expression in NAc in both amoxicillin and Augmentin groups compared to ethanol vehicle (saline) group. **(B)** Statistical analysis revealed significant upregulation of GLT-1b expression in PFC in Augmentin-treated group compared to ethanol vehicle group. However, no significant difference of GLT-1b expression in PFC was observed between amoxicillin-treated group and ethanol vehicle group. Data are shown as mean ± SEM, (^*^p < 0.05); (*n* = 5 for each group).

Statistical analysis of GLT-1a and GLT-1b immunoblots in NAc using one-way ANOVA followed by Newman-Keuls multiple-comparison *post-hoc* test revealed a significant upregulation of GLT-1a [*F*_(2, 12)_ = 5.061, *p* = 0.0255, Figure 4A] and GLT-1b [*F*_(2, 12)_ = 5.281, *p* = 0.0226, Figure 5A] in both amoxicillin and Augmentin groups as compared to ethanol vehicle group. In addition, statistical analysis of GLT-1a and GLT-1b immunoblots in PFC revealed a significant upregulation of GLT-1a [*F*_(2, 12)_= 5.957, *p* = 0.0160, Figure 4B] and GLT-1b [*F*_(2, 12)_= 5.320, *p* = 0.0222, Figure 5B] in Augmentin group only as compared to ethanol vehicle group.

**Figure 6:**
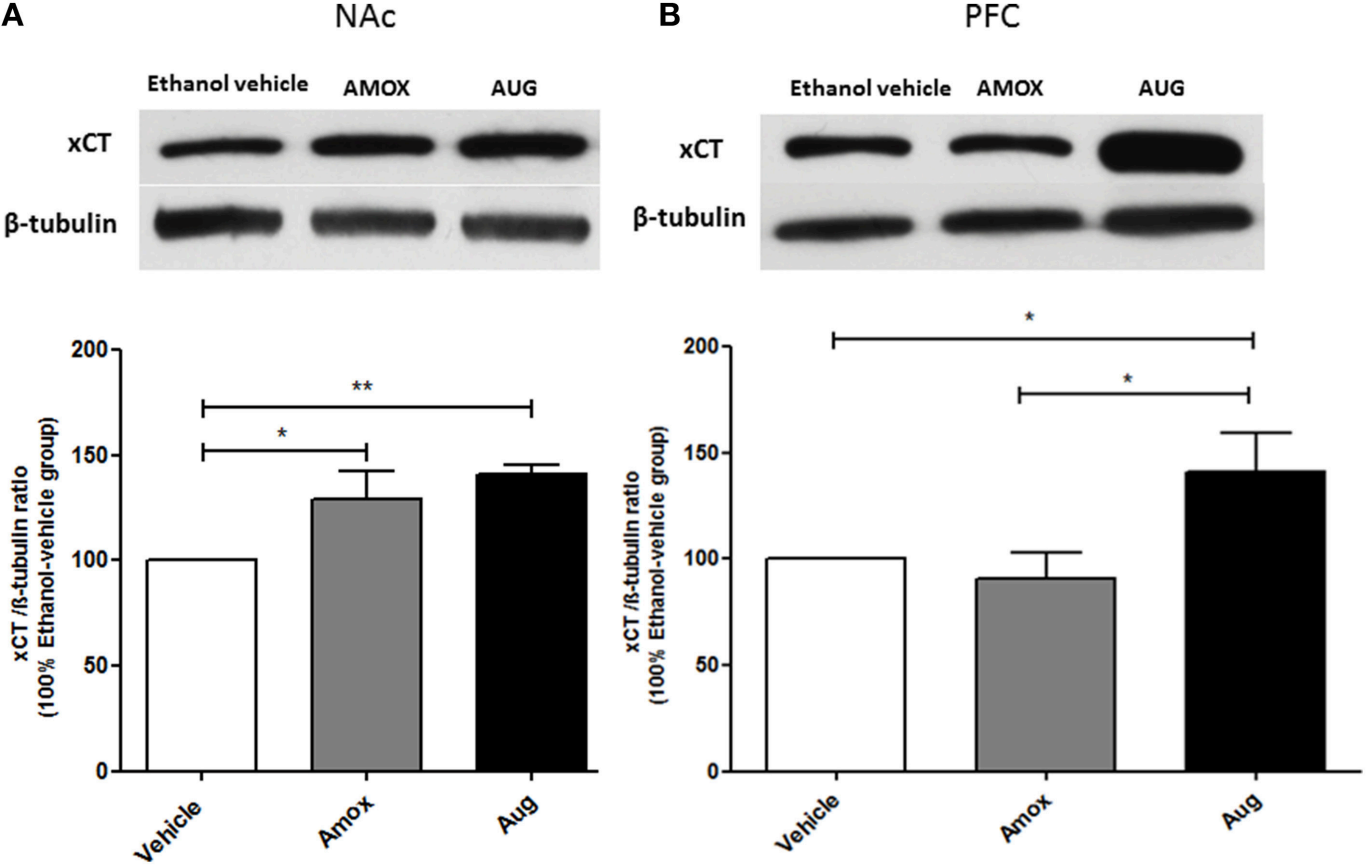
**Effects of amoxicillin (AMOX) and Augmentin (AUG) on xCT expression in NAc and PFC. (A)** Statistical analysis showed significant upregulation of xCT expression in NAc in both amoxicillin and Augmentin groups compared to ethanol vehicle (saline) group. **(B)** Statistical analysis revealed significant upregulation of xCT expression in PFC in Augmentin-treated group compared to amoxicillin-treated group and ethanol vehicle group. However, no significant difference of xCT expression was observed between amoxicillin-treated group and ethanol vehicle group. Data are shown as mean ± SEM; (^*^p < 0.05, ^**^p < 0.01); (*n* = 5 for each group).

**Figure 7:**
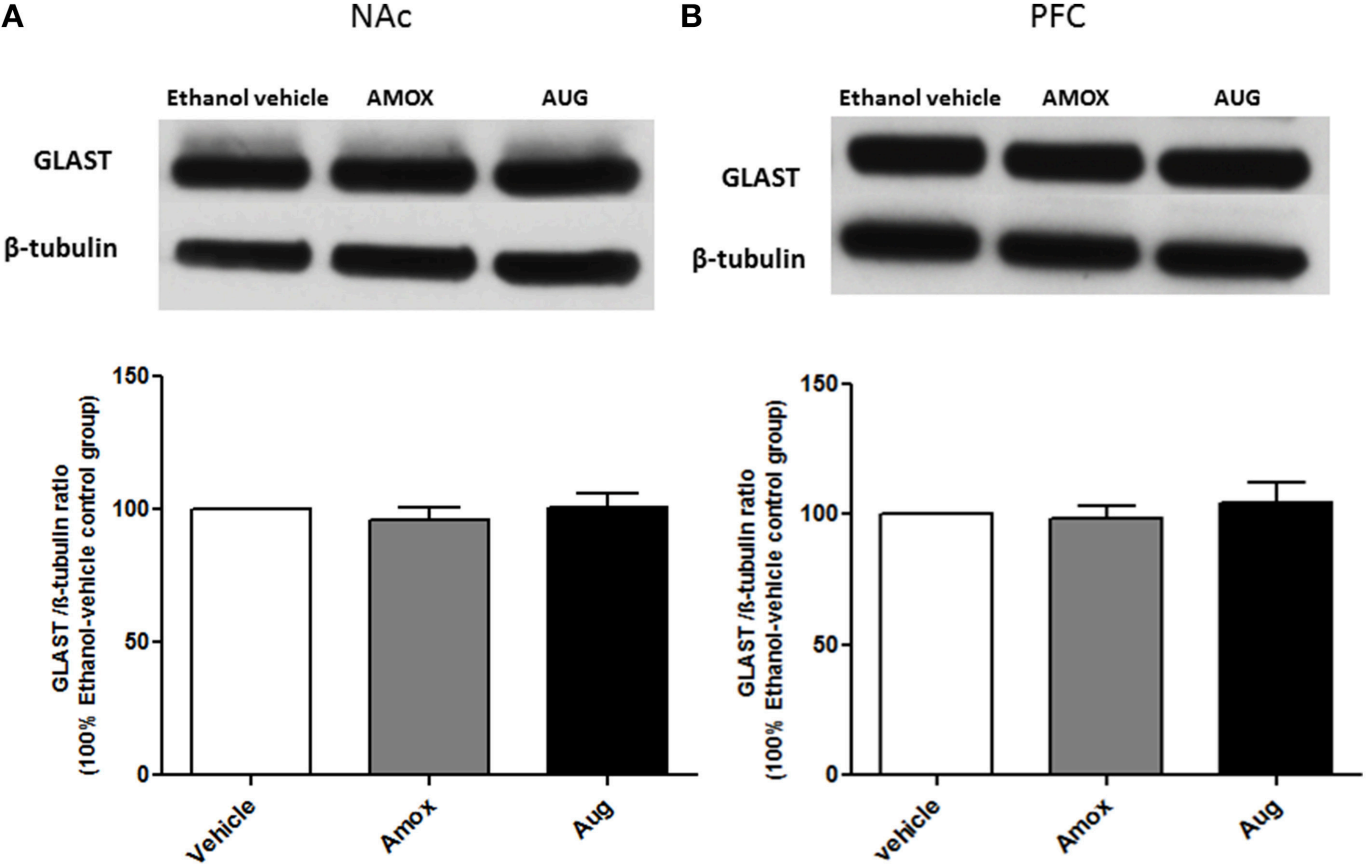
**Effects of amoxicillin (AMOX) and Augmentin (AUG) on GLAST expression in NAc and PFC. (A)** Statistical analysis showed no significant difference of GLAST expression in NAc in both amoxicillin and Augmentin groups compared to ethanol vehicle (saline) group. **(B)** Statistical analysis also revealed no significant difference of GLAST expression in PFC in both amoxicillin and Augmentin groups compared to ethanol vehicle group. Data are shown as mean ± SEM; (*n* = 5−6 for each group).

One-way ANOVA followed by Newman-Keuls multiple-comparison *post-hoc* test revealed a significant upregulation of xCT expression in both amoxicillin and Augmentin treatment groups as compared to ethanol vehicle group in NAc (Figure 6A) [*F*_(2, 12)_ = 6.821, *p* = 0.0105]. Furthermore, statistical analysis of xCT expression in PFC did not reveal any significant increase with amoxicillin-treated group as compared to ethanol vehicle group. However, Augmentin treatment revealed a significant upregulation of xCT expression in PFC as compared to ethanol vehicle and amoxicillin groups (Figure 6B) [*F*_(2, 12)_ = 4.287, *p* = 0.0394]. In addition, statistical analysis of GLAST immunoblots didn't reveal any significant difference between ethanol vehicle and treatment groups neither in NAc [*F*_(2, 15)_ = 0.4441, *p* = 0.6534] (Figure 7A) nor in PFC [*F*_(2, 15)_ = 0.5970, *p* = 0.5732] (Figure 7B).

## Discussion

This study revealed several findings as follows; (a) chronic ethanol consumption significantly reduced the expression of GLT-1 isoforms in NAc but not in PFC in male P rats, as well as reduced xCT expression in both NAc and PFC; (b) chronic ethanol exposure did not induce any changes in GLAST expression between ethanol naïve and ethanol vehicle groups; (c) administration of amoxicillin and Augmentin significantly reduced ethanol consumption and increased water intake from Day 1 through Day 5 of the treatment period as compared to ethanol vehicle group; (d) amoxicillin and Augmentin treatments increased GLT-1 isoforms and xCT expression in NAc as compared to ethanol vehicle group; (e) Augmentin, but not amoxicillin, increased GLT-1 isoforms and xCT expression in PFC as compared to ethanol vehicle group; and (f) amoxicillin and Augmentin treatments did not exhibit any changes in GLAST expression between ethanol vehicle and treatment groups.

Studies demonstrated that ethanol exposure can lead to increase extracellular glutamate concentration in NAc in mouse models (Kapasova and Szumlinski, 2008; Griffin Iii et al., 2014). Similarly, studies from ours and others demonstrated that ethanol exposure can also lead to increase in extracellular glutamate concentration in mesolimbic system, including NAc in rat models (Moghaddam and Bolinao, 1994; Melendez et al., 2005; Ding et al., 2013; Das et al., 2015). We suggested that increase in extracellular glutamate concentration might be associated in part with reduction in GLT-1 expression in NAc (Das et al., 2015). In the present study, we reported for the first time that chronic ethanol consumption decreased GLT-1a and GLT-1b expression in NAc. We also demonstrated that ethanol exposure decreased xCT expression in NAc and PFC. Downregulation of these isoforms alter neuronal and astrocytic glutamate homeostasis, which may have a critical role in ethanol intake. Astrocytes are responsible for removing the majority of extracellular glutamate concentration (Holmseth et al., 2012; Petr et al., 2015). Glutamate uptake is regulated mainly by glial GLT-1, which transports extracellular glutamate into astrocytes and then converts it to glutamine (Reviewed by ref. Danbolt, 2001). Glutamine might be then released to extrasynaptic cleft and further converted to glutamate by neurons. This glutamine-glutamate cycle is the major mechanism to recycle glutamate. Further studies are warranted to determine the role of neuronal and astrocytic glutamate uptake in ethanol craving.

It is important to note that i.p. injections of ethanol for seven days increased extracellular glutamate concentration in NAc, this effect was not associated with the reduction in GLT-1 or GLAST expression (Melendez et al., 2005). However, there is a possibility that ethanol exposure used in this latter study may have induced dysfunction of GLT-1, which might lead to alteration in glutamate transmission. The differential effects on GLT-1 expression between this latter study and ours might be due to the use of different ethanol drinking paradigms and animal models tested for determination of any changes in extracellular glutamate concentration and GLT-1 expression. Importantly, upregulation of GLT-1 expression with ceftriaxone attenuated ethanol intake and cocaine-seeking behavior was associated, in part, with restoration of extracellular glutamate concentration in NAc in rats (Sari et al., 2009; Knackstedt et al., 2010; Das et al., 2015).

Recent studies from our laboratory showed that ceftriaxone treatment increased the expression of GLT-1 isoforms and xCT in NAc and PFC and attenuated ethanol intake in male P rats (Alhaddad et al., 2014a; Rao et al., 2015). In addition, treatment with other β-lactam antibiotics (cefazolin, cefoperazone, and ampicillin) reduced ethanol consumption and induced upregulation of GLT-1 isoforms and xCT expression in NAc and PFC (Alasmari et al., 2015, 2016). In the present study, we revealed that chronic ethanol consumption decreased GLT-1 isoforms and xCT expression in NAc of male P rats. Importantly, administration of Augmentin reduced ethanol intake and upregulated GLT-1 isoforms and xCT expression in NAc and PFC; however, amoxicillin reduced ethanol intake but upregulated GLT-1 isoforms and xCT expression only in NAc. The differential effects of Augmentin vs. amoxicillin on GLT-1 isoforms and xCT might be due to the fact that Augmentin contains clavulanic acid. Thus, clavulanic acid might have a synergistic effect with amoxicillin on GLT-1 isoforms and xCT expression in NAc and PFC. It is important to note that administration of clavulanic acid upregulated GLT-1 and decreased the reinforcing effect of cocaine in mice (Kim et al., 2015). Although, amoxicillin treatment upregulated GLT-1 isoforms and xCT in NAc but not in PFC, the drug was found to reduce ethanol intake in male P rats. There is a possibility that amoxicillin may increase the activity of GLT-1 isoforms in PFC. Importantly, we demonstrated that ceftriaxone at lower dose reduced ethanol intake but did not induce any upregulatory effects in NAc and PFC (Sari et al., 2011). Previous study showed that ceftriaxone increased glutamate uptake in hippocampus without upregulating GLT-1 expression (Lipski et al., 2007). Furthermore, study from our laboratory demonstrated that MS-153 [(R)-(-)-5-methyl-1-nicotinoyl-2-pyrazoline] reduced ethanol consumption and upregulated GLT-1 expression in NAc but not in PFC (Alhaddad et al., 2014b). These data are in accordance with our present findings with amoxicillin. The lack of upregulatory effect in GLT-1 isoforms with these drugs in the PFC might be due to the fact that ethanol did not downregulate GLT-1 expression in this brain region. It is important to note that amoxicillin as well as other β-lactams may either increase the function of GLT-1 isoforms or may have other pharmacological effects that lead to reduction in ethanol intake. Studies are warranted to determine the effects of amoxicillin on glutamate uptake and extracellular glutamate concentration in PFC.

Amoxicillin and Augmentin treatments reduced ethanol intake and increased water intake as early as Day 1 (24 h after the first i.p. injection of the drugs). This is in accordance with recent study that showed similar effect with ceftriaxone (Rao et al., 2015). This latter study revealed that ceftriaxone upregulated GLT-1a and GLT-1b in PFC 48 h after the first i.p. injection with the drug. This study also demonstrated that 5 days treatment with ceftriaxone increased GLT-1 isoforms and xCT in both PFC and NAc. The reduction of ethanol intake found as early as Day 1 with amoxicillin and Augmentin might be due either to functional increase in GLT-1 isoforms or other unknown pharmacological effects that are warranted further investigation. It is important to note that our findings that demonstrated increase in water intake are in accordance with other studies using different β-lactam antibiotics (Sari et al., 2011, 2013; Rao and Sari, 2014; Alasmari et al., 2015, 2016; Rao et al., 2015). We suggest that this increase in water intake is a compensatory mechanism by the P rats in order to maintain their fluid intake. This compensatory mechanism of fluid intake might also be compared to the increase in water intake that was observed by the selectively bred high alcohol-consuming rat model during the short ethanol deprivation intervals (Bell et al., 2008).

Amoxicillin and Augmentin treatments revealed no significant change in GLAST expression between ethanol vehicle and treatment groups in NAc and PFC. These results are in consistent with previous studies from our laboratory showing that other β-lactam antibiotics did not affect GLAST expression in NAc and PFC (Alasmari et al., 2015, 2016). GLAST is highly expressed in the cerebellum to regulate glutamate homeostasis as compared to forebrain regions such as PFC and NAc, which highly express GLT-1 (Lehre and Danbolt, 1998; Bauer et al., 2012; Whitelaw and Robinson, 2013).

In conclusion, we report that amoxicillin and Augmentin treatments reduced ethanol consumption in male P rats, in part, through upregulation of GLT-1a, GLT-1b, and xCT expression in NAc and PFC. The upregulatory effects of Augmentin on GLT-1a, GLT-1b, and xCT in both NAc and PFC may suggest that this drug is more effective than amoxicillin. However, amoxicillin did upregulate GLT-1 isoforms and xCT only in NAc and yet the drug reduced ethanol intake. We suggest here that amoxicillin treatment may either lead to an increase in the activity of these glial glutamate transporters or involves other pharmacological effects on ethanol intake. This hypothesis may apply to Augmentin as well. In accordance with our previous findings, the present study supports the rationale that both amoxicillin and Augmentin decrease ethanol consumption putatively via restoration of glutamate homeostasis and other potential pharmacological effects in the central brain reward regions. Further studies are warranted to determine potential pharmacological effects involving these β-lactam antibiotics for the attenuation of ethanol dependence.

## Author contributions

AYH Participated in study design and conceptualization, drafted and revised the manuscript, collected and analyzed data, and helped with data interpretation. AH—Helped with the writing, editing, data collection, analysis, and interpretation, and approved final version of the manuscript. YS—Conceptualized and designed the study, critically revised the manuscript for intellectual content, and approved final version of the manuscript.

## Funding

This work was supported by Award Number R01AA019458 (Y.S.) from the National Institutes on Alcohol Abuse and Alcoholism.

### Conflict of interest statement

The authors declare that the research was conducted in the absence of any commercial or financial relationships that could be construed as a potential conflict of interest.
